# The Characteristics of Air Pollutants during Two Distinct Episodes of Fireworks Burning in a Valley City of North China

**DOI:** 10.1371/journal.pone.0168297

**Published:** 2017-01-03

**Authors:** Yang Song, Xiaoming Wan, Shuoxin Bai, Dong Guo, Ci Ren, Yu Zeng, Yirui Li, Xuewen Li

**Affiliations:** 1 Department of Environment and Health, School of Public Health, Shandong University, Jinan, China; 2 Institute of Geographic Sciences and Natural Resources Research, Chinese Academy of Sciences, Beijing, China; The Ohio State University, UNITED STATES

## Abstract

**Background:**

The elevation and dissipation of pollutants after the ignition of fireworks in different functional areas of a valley city were investigated.

**Methods:**

The Air Quality Index (AQI) as well as inter-day and intra-day concentrations of various air pollutants (PM_10_, PM_2.5_, SO_2_, NO_2_, CO, O_3_) were measured during two episodes that took place during Chinese New Year festivities.

**Results:**

For the special terrain of Jinan, the mean concentrations of pollutants increased sharply within 2–4 h of the firework displays, and concentrations were 4–6 times higher than the usual levels. It took 2–3 d for the pollutants to dissipate to background levels. Compared to Preliminary Eve (more fireworks are ignited on New Year’s Eve, but the amounts of other human activities are also lesser), the primary pollutants PM_2.5_, PM_10_, and CO reached higher concentrations on New Year’s Eve, and the highest concentrations of these pollutants were detected in living quarters. All areas suffered from serious pollution problems on New Year’s Eve (rural = urban for PM_10_, but rural > urban for PM_2.5_). However, SO_2_ and NO_2_ levels were 20%–60% lower in living quarters and industrial areas compared to the levels in these same areas on Preliminary Eve. In contrast to the other pollutants, O_3_ concentrations fell instead of rising with the firework displays.

**Conclusion:**

Interactions between firework displays and other human activities caused different change trends of pollutants. PM_2.5_ and PM_10_ were the main pollutants, and the rural living quarter had some of the highest pollution levels.

## Introduction

With the rapid economic development and urbanization in China, air pollution has become a serious problem in recent years. Many air pollutants can cause serious health problems and have attracted a great deal of attention [1]. In particular, airborne aerosols such as sulfate, nitrate, ammonium, particulate organic matter, black carbon, and other chemical species typically exhibit high concentrations during atmospheric haze episodes, and these pollutants have been associated with adverse effects on human health such as respiratory and cardiovascular diseases [2–4]. For instance, short-term exposure to SO_2_ can cause broncho-constriction, and SO_2_ can cause serious lung tissue damage because of its long residence time and acidic nature [5]. Inhaled NO_2_ can penetrate to the small airways of the lung and also elicit a broncho-constrictive response; individuals with asthma are often much more susceptible to these effects of SO_2_ and NO_2_ than healthy individuals [6]. Total suspended particulates (TSPs) and specifically small sized particulate matter (PM) such as PM_10_ or smaller particles have been associated with premature death, aggravated asthma, increased hospital admissions, increased respiratory problems, and coronary heart disease [7–9].

Haze events are characterized by extensive light attenuation and are related to both meteorological conditions and pollutant levels in the atmosphere [10, 11]. Scientists are presently working to demonstrate the formation and evolution mechanisms of haze to help with the design of effective measures to reduce hazy weather [12, 13], but controlling air quality in China is still a substantial challenge [14].

Besides waste gas emissions from industry and vehicles, the ignition of fireworks can contribute to air quality problems. The burning of firecrackers is known to elevate levels of several compounds including gaseous pollutants (e.g., SO_2_, NOx, and O_3_) [14] and particulate pollutants (e.g., TSP, PM_10_, and PM_2.5_), as well as various water-soluble ions and trace metals [15–17]. In India, during firework displays, the daily level of PM_10_ was found to be 2–6 times higher than the usual levels, and SO_2_ increased by nearly 10-fold [18]. In Italy, with a 4 h time resolution, PM_10_ increased to 33.6 μg/m^3^ during firework displays [19]. In China, some researchers have found that firework displays can cause serious air pollution problems in Beijing [20], Taiwan [21], and Jinan [17]. These studies focused mainly on the chemical components of the pollutants and the temporal changes in their levels, while few have investigated the ambient air quality in different functional areas of a valley city with different amounts of firework burning. This information is vital to take effective prevention measures.

Jinan is a provincial capital city in China, and it is surrounded by mountains to the north, south, and east. The city is actually located in a valley where it is difficult for waste emissions to dissipate. In this study, different pollutants were measured in Jinan during the Chinese New Year throughout two episodes of firework burning with the aim of characterizing the distribution and dissipation of pollutants in the entire city as well as within different functional areas.

## Methods

### Ethics statement

All the meteorological data collected at the nine monitoring sites used in this study are publically available on the internet, and no specific permissions are required to access these sites.

### Sampling sites

**Fig 1:**
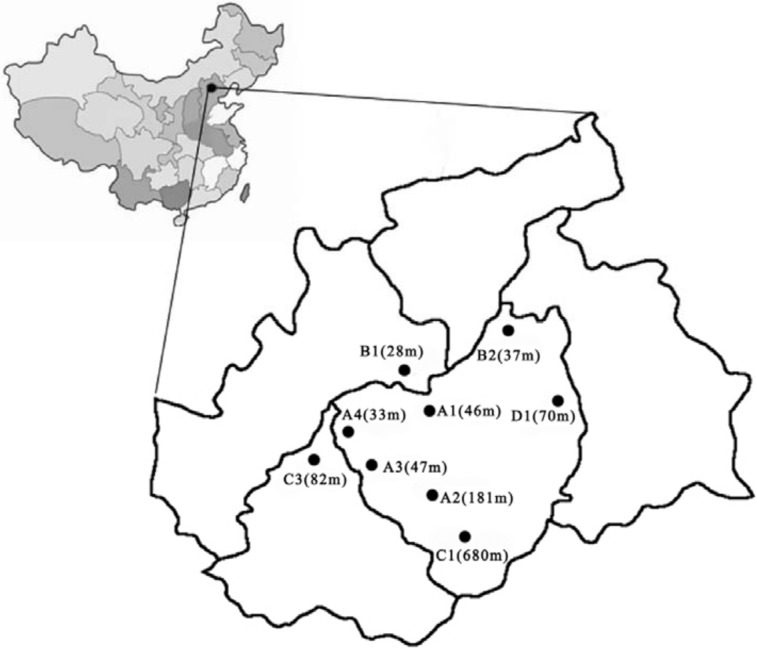
Distribution of monitoring sites in Jinan, where A1, A2, A3 are living quarters in urban areas; A4 is a living quarter in the suburbs; B1, B2 are the industrial zones in the suburbs; C1 is a tourist area in the countryside and C2 is a living quarter in the countryside; D1 is a mixed area containing living quarter and an industrial zone. Values in brackets are the elevations.

Jinan (36°40'N, 117°E) has a population of more than 7 million and an area of 8177 km^2^. There were nine monitoring sites located in different directions at a height of 15 m above the ground (Fig 1). Sites A1, A2, and A3 were located in urban residential areas, and site A4 was located in a suburban residential area; sites B1 and B2 represent the industrial areas in the suburbs. Sites C1 and C2 were located in the countryside; C1 is a tourist area and C2 is a living quarter. Site D1 consisted of a mixed residential and industrial area.

### Data collection and measurements

The days of Preliminary Eve (January 23) and New Year’s Eve (January 31) are two common periods for firework displays. The monitoring time was divided into the following five periods: before Preliminary Eve (I: January 21–22), during Preliminary Eve (II: January 23–26), after Preliminary Eve and before New Year’s Eve (III: January 27–29), during New Year’s Eve (IV: January 30 to February 3), and after New Year’s Eve (V: February 4–8). The inter-day concentrations represent the mean concentrations of pollutants during each day, and these data were collected from January 21 to February 8, 2014. The intra-day concentrations were measured every 2 h during the 2 d with firework displays.

PM_2.5_ and PM_10_ were collected on Whatman^®^ 41 filters (Whatman Inc., Maidstone, UK) every 2 h by a medium-volume sampler (model: (TSP/PM10/PM2.5)-2, flow rate: 77.59 L min^-1^). The samples were placed into polyethylene plastic bags immediately after sampling and stored in a refrigerator. All filters were weighed before and after sampling with an analytical balance (Sartorius 2004MP, reading precision of 10 μg) after stabilizing the material under constant temperature (20 ± 1°C) and humidity (40 ± 2%) conditions for over 24 h. The differences in the two totals (i.e., before and after sampling weights) were divided by the collected sampling volumes to obtain the corresponding concentrations [22]. Ozone was measured every 2 h by using a Model 49i Ozone Analyzer (Thermo Fisher Scientific, Co., Ltd) along with an ultraviolet (UV) photometer, which can accurately and reliably measure ozone concentrations in ambient air [23]. Concentrations of NO_2_, SO_2_, and CO were also measured every 2 h by gas analyzers (Thermo Fisher Scientific Inc., Franklin, MA; Model 42i, Model 43i, and Model 48i, respectively). All gas analyzers were calibrated weekly.

The diurnal concentrations of PM_2.5_, PM_10_, SO_2_, NO_2_, CO, and O_3_ were collected from http://www.tianqihoubao.com, and these data were measured by integrating with the line source long-term mean concentration model to calculate long-term mean concentrations of air pollutants from the area sources. Meteorological data such as temperature, relative humidity (RH), wind, visibility, and dew point were obtained from http://cdc.nmic.cn/home.do and WunderMap (http://wunderground.com). There was no rain from January 18 to February 8, and the wind grading was less than or equal to level 4.

The concentrations of pollutants during the festival were compared to the secondary standard reference values detailed in China’s Ambient Air Quality Standards (GB3095-1996). The reference values for SO_2_, NO_2_, PM_10_, CO, and O_3_ were 0.15 mg m^-3^, 0.08 mg m^-3^, 0.15 mg m^-3^, 10 mg m^-3^, and 0.16 mg m^-3^, respectively. The secondary standard value of the Ambient Air Quality Standards (GB3095-2012) used as the reference value for PM_2.5_ was 0.075 mg m^-3^. Measurements in excess of these reference values are indicative of air pollution.

### The Air Quality Index

The Air Quality Index (AQI) was calculated with the following formula:

$$I=\frac{I_{high}-I_{low}}{C_{high}-C_{low}}(C-C_{low})+I_{low}$$

where I = AQI, C = contaminant concentration, C_low_ = limited value less than or equal to C, C_high_ = limited value higher than or equal to C, I_low_ = limited index of AQI corresponding to C_low_, and I_high_ = limited index of AQI corresponding to C_high_.

### Characteristics of burning fireworks during the Chinese New Year

Two distinct episodes (Preliminary Eve and New Year’s Eve) involving firework burning happened during the Chinese New Year. The amount of fireworks burnt on New Year’s Eve (700 t) was much higher than that on Preliminary Eve (100 t) (the amounts were calculated as approximate values based on the generation of firework garbage). Most fireworks were ignited around 20:00 on Preliminary Eve, while there were three phases of firework burning during New Year’s Eve. The first was at 12:00, the second ranged from 18:00 to 02:00 on the following day, and the third ranged from 06:00 to 08:00 on that next day. The relative degree of firework burning was in the order of the second > the first ≈ the third. While the New Year’s Eve festivities were associated with larger amounts of firework burning, there were less industrial and vehicle emissions during these times for they occurred on a holiday.

As shown in S1 Table, the two firework episodes had almost similar meteorological conditions except for the relative humidity, which was highly variable.

## Results

### Inter-day changes in the concentrations of air pollutants

**Fig 2:**
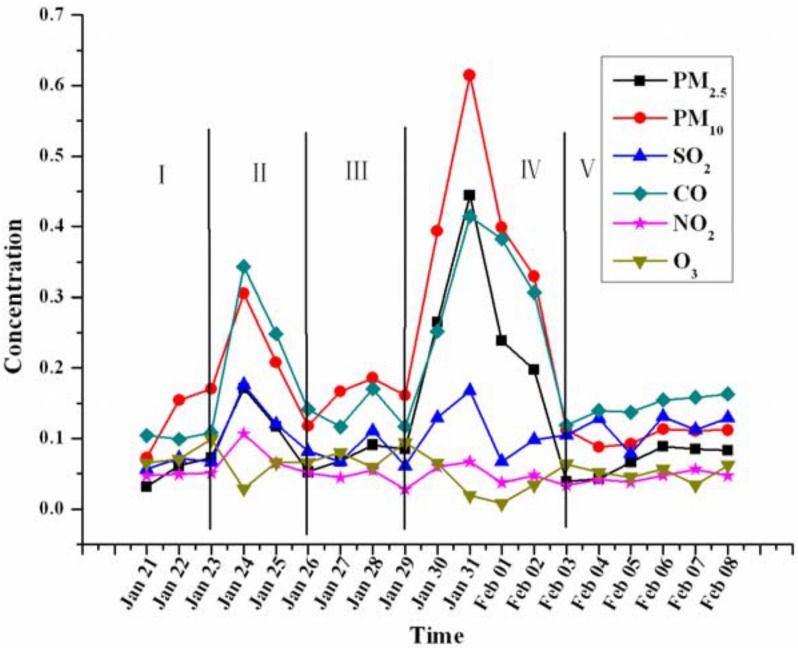
Inter-day changes of the pollutant concentrations during the two episodes for the Chinese New Year. The unit of CO is 10^−1^ mg m^-3^ and others are mg m^-3^.

The inter-day changes of the pollutant concentrations during the Chinese New Year are shown in Fig 2. There were two obvious peaks on Preliminary Eve and New Year’s Eve. The air quality was normal and stable during the periods with no firework activity (I, III, V), but during the periods of II and IV when fireworks were ignited, the concentrations of PM_2.5_, PM_10_, SO_2_, NO_2_, and CO increased sharply. The air was lightly polluted on Preliminary Eve, and the corresponding concentrations of PM_2.5_, PM_10_, CO, SO_2_, and NO_2_ were about 450%, 319%, 228%, 212%, and 125% higher, respectively, compared to the usual levels in the days before Preliminary Eve. The concentrations of PM_10_ and PM_2.5_ exceeded the reference values by 2.3 and 2.1 times respectively, and the concentrations of SO_2_ and NO_2_ were also higher than the reference values; however, the CO concentration was lower than the reference value. The concentrations of pollutants increased immediately during Preliminary Eve and recovered to normal levels within 2 d. For larger amounts of firework burning on New Year’s Eve, the concentrations of pollutants (except O_3_) were higher and it took at least 3 d for the pollutants to dissipate to background levels. The highest inter-day concentrations of PM_2.5_, PM_10_, CO, SO_2_, and NO_2_ were 520%, 381%, 354%, 273%, and 241% higher, respectively, compared to their concentrations before New Year’s Eve. The concentrations of PM_10_ and PM_2.5_ exceeded 4–6 times the reference values. The air quality was good in Period I (AQI < 100), but the AQI was 219 in Period II (heavy pollution) and 489 in Period IV (serious pollution). In all periods, the solid particulates were the primary pollutants.

### Intra-day changes in the concentrations of air pollutants

**Fig 3:**
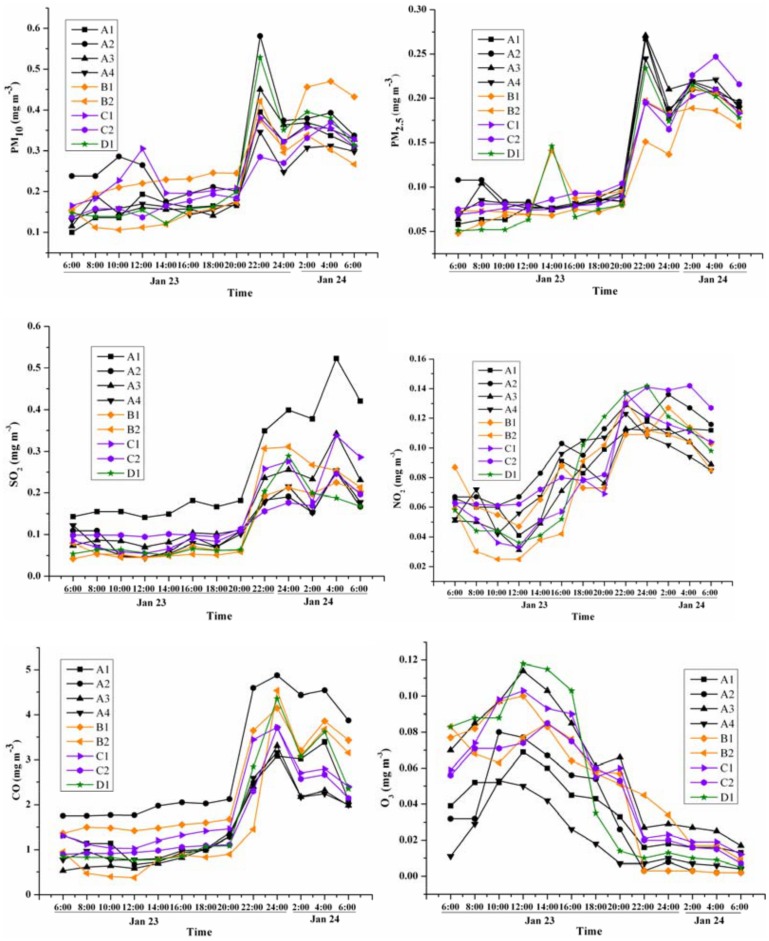
Concentration changes of pollutants at different monitoring sites before and after Preliminary Eve.

**Fig 4:**
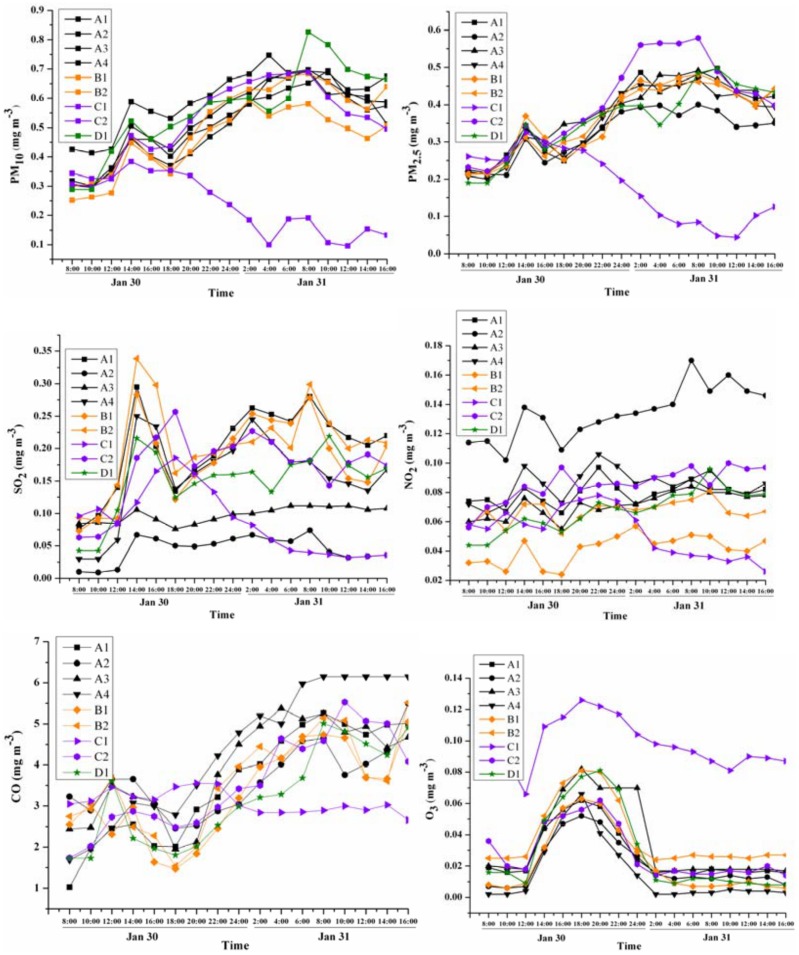
Concentration changes of pollutants at different monitoring sites on New Year’s Eve.

For more detailed information, intra-day changes of the pollutant concentrations were investigated (Figs 3 and 4). These data were separated into three categories according to the differences in trends over time on Preliminary Eve. The first category contained the solid particulates PM_2.5_ and PM_10_. These reached their highest concentrations (peak values) after 2 h of firework burning on Preliminary Eve (22:00), and concentrations were highest in the living quarters; the urban living quarters had higher levels than the rural living quarters. D1 (mixed area with living quarters and industrial activity) also had high levels of PM_10_ and PM_2.5_. The concentrations of PM_10_ and PM_2.5_ at this time exceeded the reference values by 2.3 and 2.9 times, respectively, and the levels dissipated rapidly to one-quarter and one-third of their highest concentrations, respectively, within 2 h (24:00). However, their concentrations remained high until 6:00 the next day. The second category contained the gaseous pollutants SO_2_, CO, and NO_2_. More hours (4–8 h) were needed for these pollutants to reach their peak concentrations after the firework burning. Similar changing trends for SO_2_ were observed at all sites, but the peak values of SO_2_ at different sites were highly variable. This same tendency was also observed in the NO_2_ and CO datasets. After reaching the peak levels, the concentrations began to decrease gradually and returned to normal levels after 48 h (Fig 2). The mean concentrations of SO_2_, CO, and NO_2_ at all sites exceeded the reference values by 1.7, 0.4, and 1.5 times, respectively. It should be mentioned that NO_2_ began to increase at 12:00, but there was no firework burning before this time. The third category consisted of O_3_, which changed contrary to other pollutants. Ozone was always higher in the daytime than at night, and it decreased continuously following firework burning. Generally, the degree of contamination was enhanced by firework burning and with an AQI of 210–249. The main pollutants were always PM_2.5_ and PM_10_ (Table 1). The period from 22:00 of Preliminary Eve to 6:00 of the next day was very important for the concentrations of pollutants, which reached their peak values during this timeframe and remained at high levels except for O_3_. The concentrations of PM_10_ and PM_2.5_ were 0.34 mg m^-3^ and 0.20 mg m^-3^, respectively, during this period.

**Table 1:** The Air Quality Index (AQI) and main pollutants in different sites before and after firework burning on Preliminary Eve.

Site	Before Firework Burning			After Firework Burning		
	AQI	Main Pollutant	Pollution Grade	AQI	Main Pollutant	Pollution Grade
A1	155	PM_2.5_	M	242	PM_2.5_	H
A2	165	PM_2.5_	M	244	PM_2.5_	H
A3	161	PM_2.5_	M	249	PM_2.5_	H
A4	160	PM_2.5_	M	243	PM_2.5_	H
B1	152	PM_2.5_	M	244	PM_10_	H
B2	164	PM_2.5_	M	210	PM_2.5_	H
C1	157	PM_2.5_	M	227	PM_2.5_	H
C2	162	PM_2.5_	M	243	PM_2.5_	H
D1	155	PM_2.5_	M	231	PM_2.5_	H

The time of firework burning was at 20:00. M: Moderate; H: Heavy.

The changes in pollutant concentrations on New Year’s Eve were more complicated (Fig 4); this was when larger amounts of fireworks were burnt. The concentrations of SO_2_, CO, NO_2_, and O_3_ were lower than the reference values at 08:00 on New Year’s Eve, but PM_10_ and PM_2.5_ were 0.32 mg m^-3^ and 0.22 mg m^-3^, respectively, which exceeded the reference values by 2.1 and 2.9 times, respectively. The first peak appeared at noon, with the mean concentrations of PM_2.5_, PM_10_, SO_2_, CO, and NO_2_ being 3.23, 2.34, 0.59, 0.31, and 0.79 times the reference values, respectively. After that, the concentrations of pollutants changed slightly but remained at high levels until the next day (Fig 4). During the period from 22:00 of New Year’s Eve to 6:00 of the next day, the concentrations of PM_10_ and PM_2.5_ were 0.62 mg m^-3^ and 0.45 mg m^-3^, respectively, which were about 2 times the values on Preliminary Eve. Interestingly, the concentrations of pollutants decreased at 18:00 at site C1 (tourist area).

In regard to the peak values, the levels of PM_10_ could be ranked as follows: living quarters (0.71 mg m^-3^) > industrial areas (0.64 mg m^-3^) > tourist area (0.38 mg m^-3^); no significant differences were detected between the rural living quarters and urban living quarters. The PM_2.5_ level was highest in rural living quarters (0.58 mg m^-3^). CO levels increased at all sites except C1 (tourist area). Surprisingly, with larger amounts of fireworks burnt on New Year’s Eve, the peak values of SO_2_ and NO_2_ were lower by 20%–60% in living quarters and industrial areas.

Ozone concentrations exhibited trends that were contrary to those for the other pollutants. The O_3_ concentrations increased significantly from 12:00 onward and reached peak levels at 18:00, but they decreased dramatically after the firework burning and only needed 8 h to recover to the normal, stable state. Throughout the entire monitoring period, the O_3_ concentration did not exceed the reference value (0.16 mg m^-3^).

On New Year’s Eve, living quarters had the highest AQI and the order was A2 (above 500) > A4 (431) > A3 (419) > A1 (412) (Table 2). The B sites (industrial areas) and site C2 (rural living quarters) were also seriously polluted; both had an AQI above 400. Except for site C1 (tourist area in the countryside), the pollution at most sites deteriorated during the three phases of firework burning and the main pollutants were all solid particles.

**Table 2:** The Air Quality Index (AQI) and the main pollutants during three phases of firework burning on New Year’s Eve.

Site	First Phase			Second Phase			Third Phase		
	AQI	Main Pollutant	Pollution Grade	AQI	Main Pollutant	Pollution Grade	AQI	Main Pollutant	Pollution Grade
A1	310	PM_2.5_	S	405	PM_2.5_	S	475	PM_10_	S
A2	354	PM_10_	S	>500	PM_10_	S	>500	PM_10_	S
A3	302	PM_2.5_	S	423	PM_2.5_	S	464	PM_2.5_	S
A4	294	PM_2.5_	H	444	PM_10_	S	487	PM_10_	S
B1	309	PM_2.5_	S	406	PM_2.5_	S	466	PM_2.5_	S
B2	298	PM_2.5_	H	420	PM_10_	S	>500	PM_10_	S
C1	322	PM_2.5_	S	273	PM_2.5_	H	110	PM_2.5_	M
C2	323	PM_2.5_	S	464	PM_10_	S	489	PM_2.5_	S
D1	292	PM_2.5_	H	448	PM_10_	S	500+	PM_10_	S

M: Moderate; H: Heavy; S: Serious. First Phase: 08:00–16:00 on New Year’s Eve; Second Phase: from 16:00 of New Year’s Eve to 06:00 of the following day; Third Phase: 06:00–16:00 of the following day.

## Discussion

After the firework burning, the changes in six pollutants exhibited different trends. By comparing the changes of pollutants in Figs 2, 3 and 4, the changes can be divided into three types.

The first type with a distinctive pattern included PM_10_, PM_2.5_, and CO, which increased markedly after firework burning, and their concentrations rose as more fireworks were burnt. The peak concentrations always occurred 2–4 h after firework burning and were 4–6 times higher than the usual levels (Figs 2 and 3), which is similar to the findings obtained in prior research [24]. Since the firework burning on New Year’s Eve was more intense than that on Preliminary Eve, the concentrations of PM_10_, PM_2.5_, and CO also significantly increased. Especially during the period from 22:00 on New Year’s Eve to 6:00 on the following day, the concentrations of PM_10_ and PM_2.5_ were about 2 times the values on Preliminary Eve (they increased from 0.34 mg m^-3^ to 0.62 mg m^-3^ and 0.20 mg m^-3^ to 0.45 mg m^-3^, respectively). Therefore, these changes represent a sensitive response to firework burning.

The second type with a distinctive pattern included SO_2_ and NO_2_, which did not always increase with the firework burning from Preliminary Eve to New Year’s Eve. In Figs 2 and 3, the concentrations of SO_2_ and NO_2_ increased during the period of firework burning; however, as shown in Fig 4, there were also peaks of SO_2_ and NO_2_ at 14:00 on Jan 30 after firework burning. Thus, while these data confirm that firework burning can increase the concentrations of SO_2_ and NO_2_, they also highlight that other factors are important as well. With more intense firework burning on New Year’s Eve, the concentrations of SO_2_ and NO_2_ increased slowly and the temporal response was even lower than that on Preliminary Eve. This is because on Preliminary Eve, the people were in normal living mode and human activities were stable; New Year’s Eve was a holiday and so the level of human activity differed. For example, on New Year’s Eve, except for the firework festivities, other human activities began to decrease gradually after the noon time hour, and some people left the city for reunions. Although there were large amounts of fireworks burnt in the evening, because of the lower levels of other human activities, the concentrations of SO_2_ and NO_2_ exhibited stable trends at most sites. Therefore, fireworks burning can influence the concentrations of SO_2_ and NO_2_, but these pollutants were found to be less sensitive to the effects of fireworks than the first type of pollutants.

Concentrations of NO_2_ were also influenced by other factors. In Fig 3, the time of firework burning was 20:00, but at the time from 12:00 to 20:00 when no fireworks were ignited, the level of NO_2_ increased. In addition, in the daytime on New Year’s Eve, even with more firework burning and less traffic and industrial emissions, the concentration of NO_2_ was stable. The same change of NO_2_ was also reported by Yang [17]. NO_2_ can be easily oxidized to NO_3_, thus leading to the fact that NO_3_ rather than NO_2_ is the compound predominantly found in ambient aerosols [25]. This was likely the reason that NO_2_ did not exhibit a sensitive increasing response to firework burning.

The third type consisted of O_3_. The change in O_3_ concentrations was uniquely different compared to the other pollutants measured during the festival. Ozone is produced in the atmosphere by photo-dissociation reactions involving oxygen. In this study, the concentration of O_3_ fell instead of rising during Preliminary Eve and New Year’s Eve, which indicates that the rate of O_3_ generation was less than the rate of consumption and dissipation. The highest O_3_ concentration appeared at 18:00 on New Year’s Eve and at noon on Preliminary Eve. This was because the traffic volume was normal on Preliminary Eve and NO_X_ was generated and then transformed to O_3_ by photolysis since there was sufficient sunlight. With the consumption of NO_X_ and reduced luminous intensity, O_3_ concentrations decreased gradually in the afternoon. On New Year’s Eve, the traffic volume was less and the amount of NO_X_ in the air came mainly from the firework burning. At noon on New Year’s Eve, the amount of fireworks began to increase, the concentration of NO_X_ in the air increased, and then O_3_ began to increase until the evening when the luminous intensity decreased. Meanwhile, as more fireworks were burnt at night, O_3_ decreased (to about one-third of the decreased level on Preliminary Eve); thus, these data indicate that some substances from firework displays can consume O_3_. For example, large amounts of nitric oxide (NO) produced by firework burning are strong reducing agents of O_3_. The concentration of O_3_ during the intensive period of firework burning was low. As shown in Fig 4, the tourist area (C1), which was associated with less firework burning, had higher O_3_ concentrations than the other sites; these data also provide evidence that firework burning can influence O_3_ distribution patterns. Similar trends were observed in a former study in India [26].

Spatial variations in pollutant concentrations were also evident after firework burning. On Preliminary Eve, the peak values of PM in urban and suburban living quarters were 0.474 ± 0.065 mg m^-3^ for PM_10_ and 0.261 ± 0.065 mg m^-3^ for PM_2.5_, which were higher than those in rural living quarters (p < 0.01), where the concentrations of PM_10_ and PM_2.5_ were 0.405 ± 0.116 mg m^-3^ and 0.236 ± 0.092 mg m^-3^, respectively. While on New Year’s Eve when larger amounts of fireworks were burnt, the peak concentrations of PM pollutants in urban and suburban living quarters were 0.453 ± 0.065 mg m^-3^ for PM_10_ and 0.457 ± 0.065 mg m^-3^ for PM_2.5_, which were higher than those in industrial areas (p < 0.01), at this time, the concentrations of PM_10_ and PM_2.5_ in industrial areas were 0.397 ± 0.116 mg m^-3^ and 0.364 ± 0.092 mg m^-3^, respectively. Rural living quarters showed the highest peak concentrations of PM during this time. The tourist area had the lowest level of PM. CO also reached the highest peak concentrations in living quarter areas. This was related to the firework burning, which was time-limited in urban areas but not in rural areas. A previous study in Beijing also found that the concentrations of pollutants in restricted-burning areas were much lower than those in non-restricted areas, which means the limitation of firework burning by governments can have a positive influence on air quality [27].

Most sites were seriously polluted on New Year’s Eve (Table 2). From the first phase to the third phase of firework burning, the AQI at most sites increased from 300 to 500. However, the concentrations of pollutants at C1 were the lowest, with an API ranging from moderate to serious pollution. Site C1 is a tourist area located in the southern mountains of Jinan. In the morning on New Year’s Eve, there were some visitors and stores were open as usual. Some firework burning still took place; but in the afternoon, stores closed and people went home for the festival, so firework burning decreased and the levels of pollution decreased in the afternoon.

In summary, judging from the concentration changes and AQI, PM_2.5_ and PM_10_ were the main air pollutants from firework burning. During the New Year’s festival, the sharp rise in the levels of pollutants put the air pollution in a serious state. The concentrations of PM_2.5_, PM_10_, and SO_2_ were 4–7 times higher than reference values, and people exposed to this air for more than 10 min could suffer from mild asthma like symptoms; in severe pollution situations, respiratory functions could be affected [27, 28]. The morbidity associated with asthma and bronchitis induced by exercise is apparently connected to PM_2.5_ concentrations [29], and SO_2_ concentrations have been found to be closely associated with the number of hospitalized patients with bronchial asthma, bronchitis, upper respiratory tract infections, and pneumonia; specifically, higher SO_2_ concentrations were found to be correlated with larger daily numbers of visiting patients with the above mentioned respiratory problems [30]. Studies have also revealed that PM_2.5_ exposures are associated with inflammatory damage in the lung, which can disrupt the cellular micro-environment and decrease repair mechanisms of local lung tissues [31, 32]. According to the data presented in Fig 2, Tables 1 and 2, the air quality from the start of the firework burning until 2 d later was serious polluted. Children and elderly patients with heart or pulmonary disease are more vulnerable to poor air quality, and it would be worthwhile to take corrective actions to prevent respiratory diseases in these vulnerable populations due to exposures to air pollutants [1].

In China, many people and most physicians are aware of the harmful influence of poor AQI scores, but the symptoms caused by the different pollutants are different and people may have trouble recognizing a cause and effect relationship; in fact, it is even difficult for physicians to identify the different symptoms resulting from exposures to different air pollutants, and thus, it would be worthwhile to develop a public health guidebook detailing the range of adverse effects that people may experience. Additionally, the different symptoms caused by sudden or long-term changes of pollutant concentrations should be differentiated by physicians. Physicians have an obligation to help people reduce their exposures to air pollutants by having them check the local AQI every day, and extra care should be taken with children and elderly patients as well as those with bronchial asthma and chronic bronchitis [33]. As for communities, it would be wise for residents to become familiar with the air quality regulations issued by local governments and to take corresponding measures to reduce air pollution. The government is the main institution responsible for collecting and sharing air quality information and limiting air pollution such as that caused by firework burning through strict laws and regulations. Hence, communities should cooperate with the government to provide timely and accurate information about the air quality conditions and associated regulations to the residents.

## Conclusions

This study found that temporal and spatial distributions of various pollutants can be affected by intensive firework burning. PM_2.5_ and PM_10_ were the main pollutants involved, and serious air pollution episodes were observed. Living quarters, especially in rural areas on New Year’s Eve, were associated with the highest risk. For the special terrain of Jinan (valley city), the pollutants needed more time to dissipate to normal levels. Therefore, stricter rules should be put in place here to limit firework burning in terms of the time and place.

## Supporting Information

S1 Table Diurnal meteorological conditions during the Chinese New Year. (DOCX)
