# Supplementary material for: The Characteristics of Air Pollutants during Two Distinct Episodes of Fireworks Burning in a Valley City of North China
Source: PLoS One. 2017 Jan 3;12(1):e0168297. doi: 10.1371/journal.pone.0168297 (PMC5207441; doi:10.1371/journal.pone.0168297)
Supplement: S1 Table — (DOCX) [file pone.0168297.s001.docx]

**Supporting Information**

**Table S1.** Diurnal meteorological conditions during the Chinese New Year.

| Date | Temp (°C) | RH (%) | WG | DP (°C) | Visibility  (km) |
| --- | --- | --- | --- | --- | --- |
| I |  |  |  |  |  |
| Jan 18 | 0 | 57 | <3 | -11 | 11.6 |
| Jan 19 | 4 | 36 | 3 | -10 | 6.6 |
| Jan 20 | 2 | 25 | 4 | -18 | 20.0 |
| Jan 21 | -2 | 27 | 4 | -19 | 27.6 |
| Jan 22 | 5 | 19 | 3 | -17 | 20.0 |
| II |  |  |  |  |  |
| Jan 23 | 9 | 20 | 3 | -13 | 18.6 |
| Jan 24 | 8 | 31 | <3 | -8 | 8.2 |
| III |  |  |  |  |  |
| Jan 25 | 6 | 47 | 3 | -7 | 28.0 |
| Jan 26 | 2 | 34 | 4 | -14 | 23.2 |
| Jan 27 | 9 | 36 | 4 | -6 | 19.4 |
| Jan 28 | 6 | 37 | 4 | -10 | 20.0 |
| Jan 29 | 8 | 45 | 4 | -3 | 10.2 |
| IV |  |  |  |  |  |
| Jan 30 | 7 | 72 | 3 | 2 | 2.8 |
| Jan 31 | 6 | 70 | 3 | 2 | 1.9 |
| Feb 1 | 3 | 94 | < 3 | 1 | 1.2 |
| Feb 2 | 4 | 92 | 3 | 2 | 2.1 |
| Feb 3 | 0 | 54 | 3 | -10 | 30.0 |
| Feb 4 | -2 | 33 | 4 | -17 | 30.0 |
| V |  |  |  |  |  |
| Feb 5 | -2 | 78 | 3 | -5 | 8.6 |
| Feb 6 | -2 | 84 | 4 | -4 | 7.5 |
| Feb 7 | -2 | 81 | 4 | -5 | 7.0 |
| Feb 8 | -4 | 73 | 4 | -8 | 14.2 |

Temp, Temperature; RH, Relative Humidity (%); WG, Wind Grading; DP, Dew Point.
